# Caries prevalence of the first permanent molars in 6–8 years old children

**DOI:** 10.1371/journal.pone.0245345

**Published:** 2021-01-13

**Authors:** Fudong Zhu, Yao Chen, Yunxian Yu, Yanyi Xie, Haihua Zhu, Huiming Wang

**Affiliations:** 1 The Affiliated Hospital of Stomatology, School of Stomatology, Zhejiang University School of Medicine, Hangzhou, Zhejiang, China; 2 Key Laboratory of Oral Biomedical Research of Zhejiang Province, Hangzhou, Zhejiang, China; 3 School of Public Health, Zhejiang University, Hangzhou, Zhejiang, China; Centre Hospitalier Regional Universitaire de Tours, FRANCE

## Abstract

Dental caries is one of the most common infectious diseases affecting 6–8-year-old children, especially their first permanent molars (FPMs). This study explored the prevalence of dental caries on FPMs by analyzing the oral health status of 1,423,720 children aged 6–8 years in Zhejiang Province, China. The data were extracted from the dental electronic records of the schoolchildren attending the Oral Health Promotion Project (OHPP), conducted during 2013–2017 in Zhejiang Province. Multiple logistic regression models were used to determine the factors affecting dental caries. Boys and girls accounted for 53.2% and 46.8% of the subjects, respectively. From 2013 to 2017, the prevalence of dental caries on FPMs increased: 2013: 20.4%; 2014: 25.3%; 2015: 24.5%; 2016: 27.0%; and 2017: 29.0%, despite the OHPP conducted. Based on multiple logistic regression model, girls had a significantly higher risk of FPM caries compared to boys (OR = 1.38, 95% CI: 1.37–1.39, *p* < 0.0001); compared with the caries rates in urban areas, the caries risk was significantly higher in rural areas (OR = 1.15, 95% CI: 1.14–1.16, *p* < 0.0001). In terms of geographic location in Zhejiang Province, the odds ratios of the caries risk of the east, south, west, and north were 1.35 (1.33–1.36), 1.3 (1.28–1.31), 0.81 (0.8–0.83), and 0.82 (0.81–0.84), respectively (*p* < 0.0001), by considering the central region as a reference. The caries prevalence of FPMs was high, with an increasing tendency and gender, social, cultural, and environmental factors affecting the caries prevalence.

## Introduction

Dental caries is one of the most prevalent infectious diseases all over the world, and individuals are susceptible to this condition throughout their whole lifetime [1]. Among 5–17-year-old Americans, it is more than five times as common as reported asthma and seven times as common as hay fever [2]. Data from the 4th Chinese National Oral Health Survey (CNOHS) show that the prevalence of dental caries has increased over the past 10 years in children from 5 to 12 years of age [3]. What’s more, obvious differences are discovered between boys and girls in Zhejiang province, with the prevalence 38.5% and DMFT 0.92 in 12 years old boys and the prevalence 51.0% and DMFT 1.49 in 12 years old girls [4].

World Health Organization (WHO), World Dental Federation (FDI), and International Association for Dental Research (IADR) suggested that specific values should be considered “with regard of the political, socioeconomic, and legislative context,” leaving the decision about the definite aims of caries prevention programs to the national level in 2003 [5]. Thus, according to the *‘Healthy China 2030’* blueprint, one of the aims of caries prevalence programs in China is to reduce dental caries to <25% of the 12-year-old children in 2030.

The first permanent molar (FPM) is the most common tooth in children, which are affected by dental caries. FPMs have been reported to be highly susceptible to caries attack [6]. The patients with high caries experience in early life exhibit an increased risk of caries in adulthood [7]. Furthermore, a one-unit increase in the number of carious first molars was associated with a significant increase in the number of other carious teeth [8]. FPMs are very important because of their significant role in maintaining a normal masticatory function and dentofacial harmony [9]; therefore, their effect on the overall development of children cannot be overemphasized. However, it is the very lack of visibility for molars compared to the anterior teeth, combined with the increased difficulty of cleaning the posterior interdental spaces, which increases the caries risk of FPMs. Therefore, the prevention of FPM caries plays a critical role during childhood [10].

As a result, the OHPP, which encompasses oral health education and pit and fissure sealants for FPMs to prevent FPM caries in China, has been sponsored by the government since 2009 in Zhejiang Province, located in southeast China, with coasts, open plains, and hills. As coastal areas, the local economy is more developed in the north (Hangzhou, Jiaxing, Huzhou), east (Shaoxing, Ningbo, Zhoushan) and south (Wenzhou, Taizhou, Lishui) part of Zhejiang than in the central (Jinhua) and west (Quzhou) part. The dietary pattern of residents in coastal areas is mostly similar to the Western dietary pattern. Those living in the open plains prefer traditional southern Chinese dietary; and those living in the hills eat more grains and vegetables [11]. It is a representative sample of Chinese provinces. In order to improve children’s oral health, a series of oral health measures was launched for second-year schoolchildren, and a system was designed for the management of children’s oral health. All the data related to the FPM of 6–8-year-old students were collected in almost all the schools in Zhejiang Province from 2013 to 2017 to investigate the FPM caries epidemiology systematically. An attempt was made to figure out the FPM caries risk pattern concerning gender, calender, region and urban/rural in the last five years and determine the relevant risk factors to provide essential data for formulating and optimizing prophylaxis policies for dental caries, especially in adolescents, in the future.

## Materials and methods

This study conducted in 2019 included all the available records in the electronic system of the OHPP during 2013–2017. With written informed consent distributed by class teachers and then recovered from guardian participants over the years, all the second grade schoolchildren aged 6–8 from all the primary schools in the province underwent clinical examinations of their FPMs and, if suitable, accepted pit and fissure sealing. The project was approved by the Stomatological Ethics Committee of the Chinese Stomatological Association and the Ethics Committee of Stomatology Hospital Affiliated to Zhejiang University School of Medicine (No.2014-003).

A total of 86,762 volunteer dentists participated in the project for five years after they were provided with a brochure with guidelines for clinical examinations and detection of caries based on WHO criteria [12]. All these dentists were trained step by step from provincial level to municipal level and county level. Each year, at least 2 districts or counties would be selected randomly to receive conduct and supervision from the provincial group, which was composed of all the authors. The inspectors whose inter-examiner and intra-examiner reliability of kappa coefficient were less than 0.8 could not pass the standard consistency test and participate in the project. Intra-examiner reliability were assessed by the same examiner with the same children for 2 times. While inter-examiner reliability were assessed by different examiners with the same participants. A gold standard was used as follows. A crown was recorded as sound if it exhibited no evidence of treated or untreated dental caries. The stages of caries that preceded cavitation, as well as other conditions similar to the early stages of the caries process like dental fluorosis and enamel hypoplasia, were considered sound because they could not be reliably diagnosed. Caries was recorded as present when a lesion in the pit and fissure or on a smooth tooth surface presented as an unmistakable cavity, undermined enamel, or a detectable softened floor or wall. When in doubt, caries was not recorded as present. Detection of dental caries in the survey was performed at cavitation level because, in many cases, the examiners, in general, cannot reliably access the non-cavitated lesions without X-ray.

All the FPMs were examined under artificial light using dental mirrors and explorers and for two categorical characteristics: 1) eruption status (categorized as not erupted, partially erupted, and fully erupted); 2) caries status according to WHO criteria [12] in the fully erupted FPMs. Before the oral examinations, the children were instructed by the dentists to brush their teeth at school. The examiners dried the tooth surfaces with cotton rolls and swabs, with the students in a supine position. No radiographic examinations were performed. After the examinations, the reports were sent to the children’s caretakers to inform them if they needed treatment.

An oral health management system has been developed in Zhejiang Province to facilitate the management of children’s oral health. The data in this report came from this system. The dataset contained the following information: (1) school’s name and ID; (2) the location of the school; (3) the date of examination; (4) the socio-demographic information about the children, including age, gender, rural/urban area (defined by the administrative division); (5) the status of the first permanent molars in terms of eruption, the use of pit and fissure sealants, caries (sound, decayed, missed, filled with caries, filled with no caries), and the location of caries. Authors had access to information that could identify individual participants during or after data collection.

### Statistical analysis

A descriptive analysis was conducted using the R software (Version 3.5.2). The categorical variables were expressed as numbers and percentages. First, the caries prevalence was described in terms of the molar location. Second, the relationship was estimated between the socio-demographic characteristics and caries prevalence in each FPM, using multiple logistic regression models. The socio-demographic characteristics included calendar year (from 2013 to 2017), gender (boy or girl), area (rural or urban), and geographic location (central, east, north, west, and south). The odds ratio (OR) and its 95% confidence interval (95% CI) were generated for estimating the strength of the association between the relevant variables and outcomes. Statistical significance was set at *p* < 0.05.

## Results

### Study population

A total of 1,423,720 subjects (53.2%, male; 46.8%, female) were included in the analysis, excluding those who exhibited a lack of compliance (childern with strong vomiting reflex or poor cooperation), were absent, had no parental consent, or had incomplete records, taking a less than 10% proportion of the overall subjects.

### Evaluation of tooth eruption

Data on the eruption stage of FPMs in 1,423,720 subjects collected in 2013–2017 led to the inclusion of 5,690,922 first molar teeth in the analysis, of which 4,938,047 (86.8%) molars were categorized as fully erupted. Table 1 presents the distribution of FPMs in terms of eruption status and gender. All the teeth position were indicated with the method stated by Fédération Dentaire Internationale in 1970. Those first molars which has been lost due to severely caries would not be recorded as any of the three eruption status. Hence, the totals differed between #16, #26, #36 and #46.

**Table 1:** The distribution of FPMs according to eruption status and sex.

	Gender	#16	#26	#36	#46	Total
Non-eruption	boy	60800(57.7)	58321(57.8)	35169(63.4)	36967(63.4)	191257
	girl	44597(42.3)	42568(42.2)	20283(36.6)	21353(36.6)	128801
Partial-eruption	boy	50502(55.3)	50357(55.5)	72610(57.8)	71982(57.6)	245451
	girl	40835(44.7)	40448(44.5)	53097(42.2)	52986(42.4)	187366
Fully-eruption	boy	646257(52.7)	648792(52.7)	649558(52.3)	648327(52.3)	2592934
	girl	580021(47.3)	582351(47.3)	591893(47.7)	590848(47.7)	2345113
Total		1423012	1422837	1422610	1422463	5690922

### Distribution of caries

**Fig 1:**
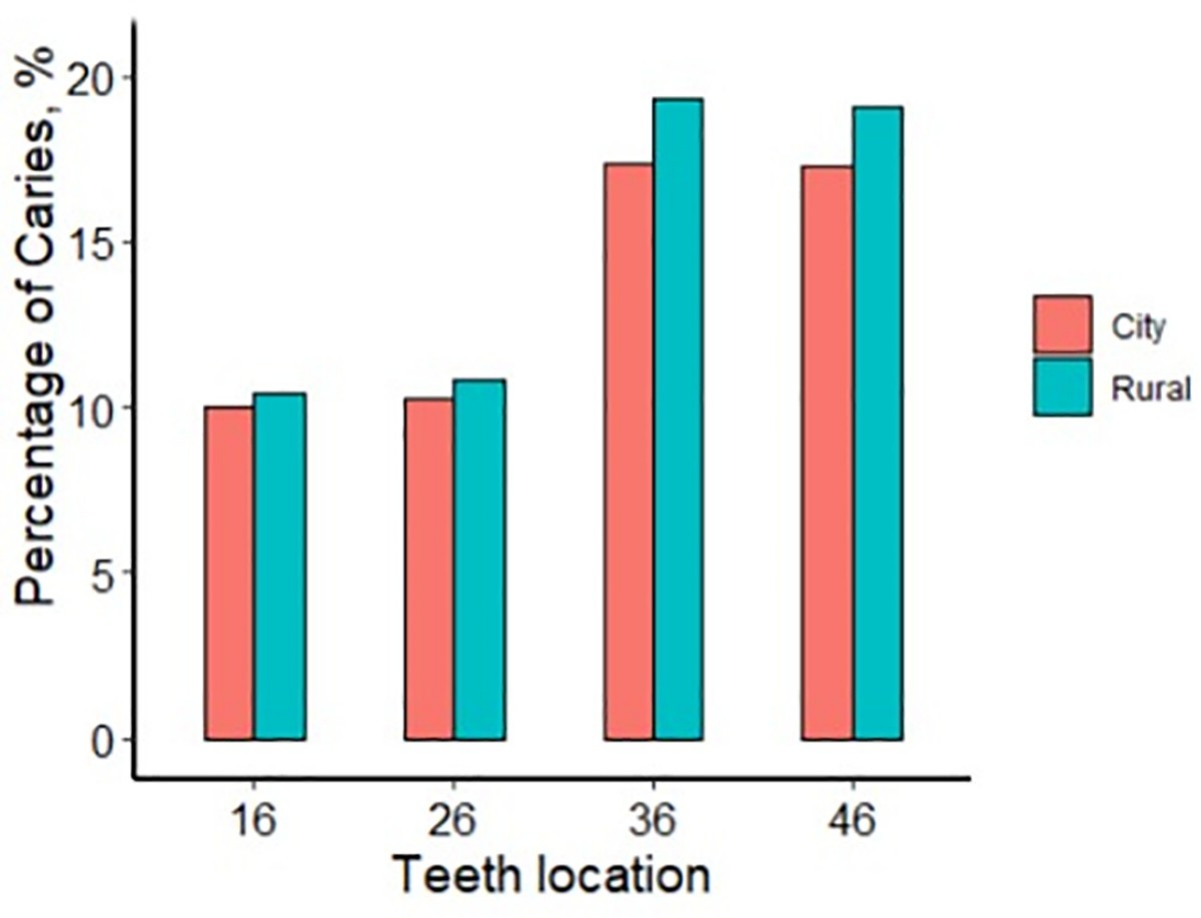
Prevalence of caries in the urban and rural area.

Carious lesions were examined and rated in all the fully erupted FPMs. Table 2 presents the distribution of carious lesions separately in terms of the calendar year, gender, rural or urban area, and geographic region of residence. The female subjects and those living in rural areas were likely to have more carious lesions. The children from the eastern and southern regions had more numerous dental caries. The proportion of female children with sound FPMs was 71.2% compared to 77.1% in male children (*p* < 0.001). From 2013 to 2017, the prevalence of FPM caries increased gradually, with 20.4% in 2013, 25.4% in 2014, 24.5% in 2015, 27.0% in 2016, and 29.0% in 2017. Among them, more than half of the children had more than one FPM caries. The percentages of the subjects with sound FPMs were 71% in the east, 71.4% in the south, 76.6% in the central region, 79.1% in the west, and 79.7% in the north. When sorted by the variables of urban or rural areas, 24.3% of students living in urban areas and 26.9% living in rural areas had FPM caries. Fig 1 presents more detailed information. For example, 10.5% of children in rural areas had caries in tooth #16 (right upper FPM) compared to 10.0% in urban children, which is significantly different (*p* < 0.001). For teeth #26, #36, and #46, caries prevalence rates were 10.3%, 17.3%, and 17.2% in rural areas, with 10.8%, 19.3%, and 19.1% in urban areas, respectively (*p* < 0.001).

**Table 2:** The distribution of caries teeth number between general variables among children.

Variable	Number of carious teeth, n (%)				
	0	1	2	3	4
**Gender**					
Boy	452279 (77.1)	44165 (7.5)	43177 (7.4)	18393 (3.1)	28328 (4.8)
Girl	380321 (71.2)	45648 (8.5)	49239 (9.2)	22419 (4.2)	36600 (6.9)
**Calendar**					
2013	53271 (79.6)	4964 (7.4)	4432 (6.6)	1713 (2.6)	2562 (3.8)
2014	229148 (74.6)	24454 (8.0)	24779 (8.1)	11036 (3.6)	17551 (5.7)
2015	244303 (75.5)	25594 (7.9)	25850 (8.0)	11005 (3.4)	16940 (5.2)
2016	208905 (73.0)	23640 (8.3)	25122 (8.8)	11136 (3.9)	17562 (6.1)
2017	96973 (71.0)	11161 (8.2)	12233 (9.0)	5922 (4.3)	10313 (7.5)
**Region**					
Middle	178376 (76.6)	17785 (7.6)	16618 (7.1)	6791 (2.9)	13238 (5.7)
East	227429 (71.0)	27293 (8.5)	30271 (9.4)	13529 (4.2)	22017 (6.9)
South	213755 (71.4)	26692 (8.9)	27676 (9.2)	13343 (4.5)	17895 (6.0)
West	57760 (79.1)	5410 (7.4)	5231 (7.2)	2100 (2.9)	2487 (3.4)
North	155280 (79.7)	12633 (6.5)	12620 (6.5)	5049 (2.6)	9291 (4.8)
**Urban/rural**					
Urban	384361(75.7)	38169 (7.5)	38587 (7.6)	17249 (3.4)	29131 (5.7)
Rural	448239 (73.1)	51644 (8.4)	53829 (8.8)	23563 (3.8)	35797 (5.8)

**Fig 2:**
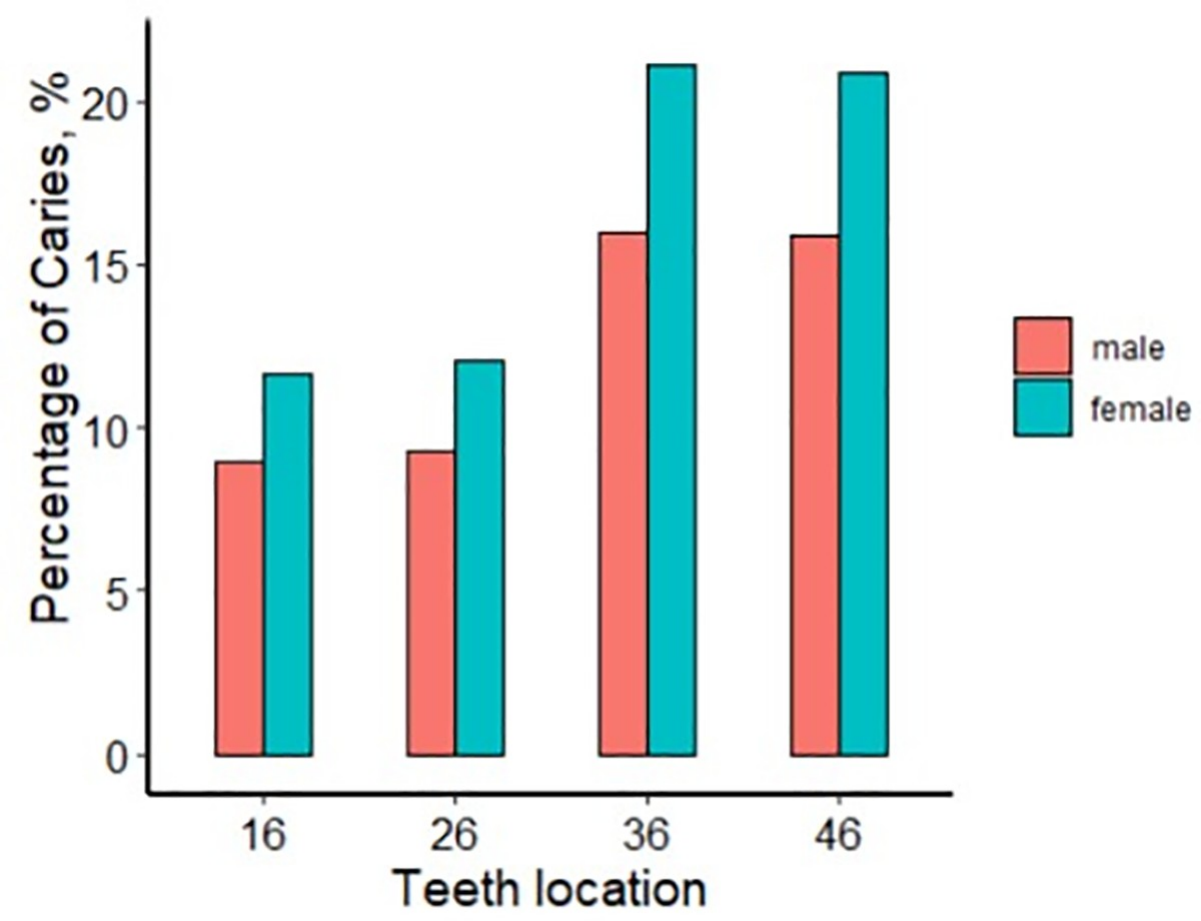
Prevalence of caries in boys and girls.

**Fig 3:**
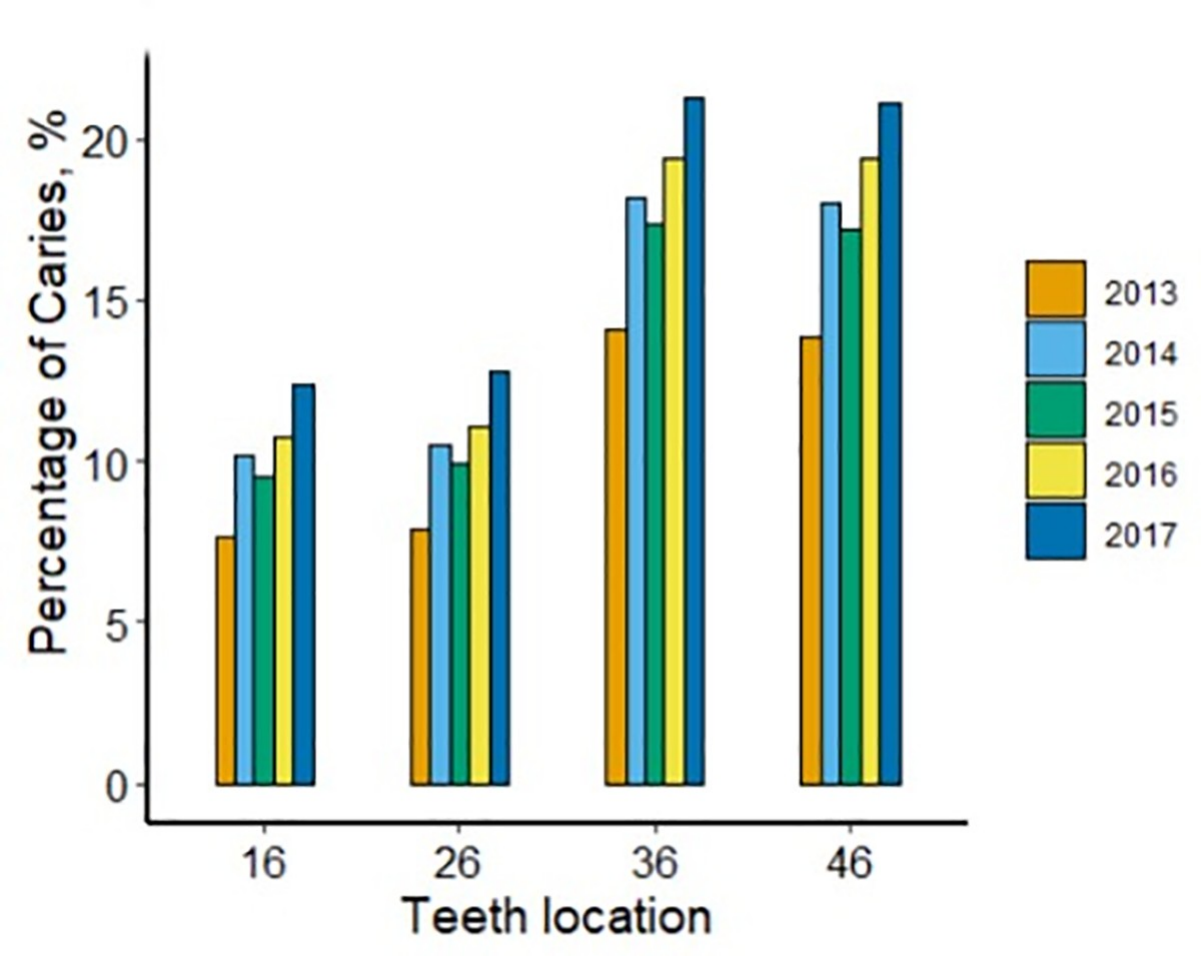
Prevalence of caries from 2013 to 2017.

**Fig 4:**
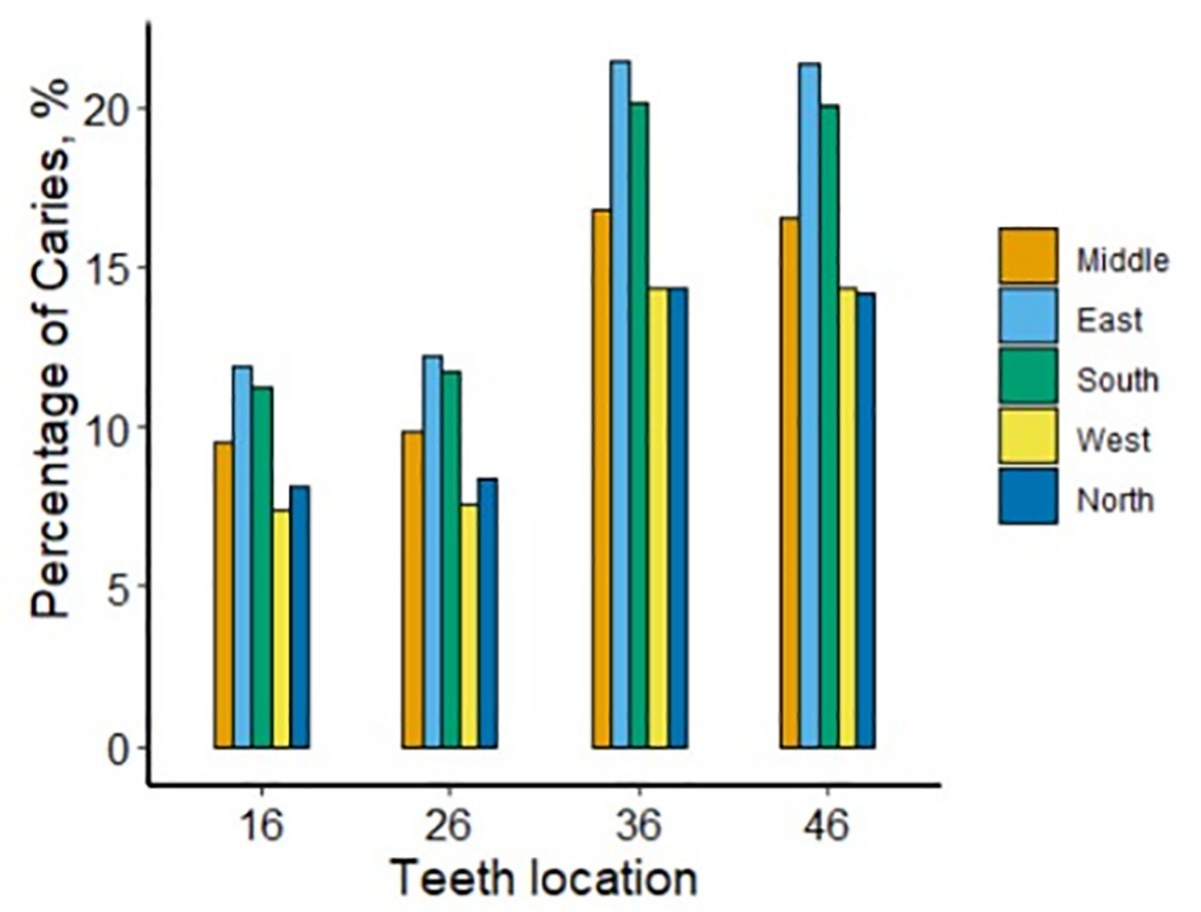
Prevalence of caries from different regions.

The distributions of caries on FPMs in different quadrants were compared in terms of gender, calendar year, region, and rural and urban areas (Table 3 and Figs 1–4), with all exhibiting statistically significant differences (*p* < 0.001). FPM caries prevalence of boy was 9% for tooth #16, 9.3% for #26, 16% for #36, and 15.9% for #46, with 11.7% for #16, 12.1% for #26, 21.1% for #36, and 20.9% for #46 in girls. The differences in FPM caries rates between boys and girls were significant (*p* < 0.001) (Fig 2).

**Table 3:** The distributions of caries between each characteristic.

Variable	Teeth	No caries	Caries	*p*
**Gender**				
**Boy**	#16	586591 (91.0)	57757 (9.0)	<0.001
	#26	586596 (90.7)	59895 (9.3)	
	#36	544399 (84.0)	103366 (16.0)	
	#46	543954 (84.1)	102518 (15.9)	
**Girl**	#16	510950 (88.3)	67544 (11.7)	<0.001
	#26	510446 (87.9)	70007 (12.1)	
	#36	465871 (78.9)	124555 (21.1)	
	#46	466229 (79.1)	123082 (20.9)	
**Calendar year**				
**2013**	#16	67573 (92.3)	5623 (7.7)	<0.001
	#26	67645 (92.1)	5821 (7.9)	
	#36	63983 (85.9)	10497 (14.1)	
	#46	63947 (86.2)	10276 (13.8)	
**2014**	#16	301498 (89.9)	34034 (10.1)	<0.001
	#26	301268 (89.5)	35228 (10.5)	
	#36	277781 (81.8)	61681 (18.2)	
	#46	277904 (82.0)	60908 (18.0)	
**2015**	#16	317933 (90.5)	33348 (9.5)	<0.001
	#26	317971 (90.1)	34921 (9.9)	
	#36	295099 (82.6)	62140 (17.4)	
	#46	295150 (82.8)	61451 (17.2)	
**2016**	#16	279273(89.2)	33711 (10.8)	<0.001
	#26	279519(88.9)	34803 (11.1)	
	#36	254856(80.6)	61507 (19.4)	
	#46	254600(80.6)	61248(19.4)	
**2017**	#16	131264 (87.6)	18585 (12.4)	<0.001
	#26	130639 (87.2)	19129 (12.8)	
	#36	118551 (78.7)	32096 (21.3)	
	#46	118582 (78.9)	31717 (21.1)	
**Region**				
**Middle**	#16	229165 (90.5)	24186 (9.5)	<0.001
	#26	229202 (90.2)	24976 (9.8)	
	#36	212917 (83.2)	42935 (16.8)	
	#46	213276 (83.5)	42198 (16.5)	
**East**	#16	309603 (88.1)	41628 (11.9)	<0.001
	#26	309852 (87.8)	43054 (12.2)	
	#36	277842 (78.6)	75817 (21.4)	
	#46	277430 (78.7)	75203 (21.3)	
**South**	#16	284235 (88.8)	35874 (11.2)	<0.001
	#26	283257 (88.3)	37568 (11.7)	
	#36	259409 (79.8)	65570 (20.2)	
	#46	259373 (79.9)	65169 (20.1)	
**West**	#16	74257 (92.6)	5909 (7.4)	<0.001
	#26	74340 (92.5)	6056 (7.5)	
	#36	71263 (85.7)	11937 (14.3)	
	#46	71214 (85.7)	11885 (14.3)	
**North**	#16	200281 (91.9)	17704 (8.1)	<0.001
	#26	200391 (91.7)	18248 (8.3)	
	#36	188839 (85.6)	31662 (14.4)	
	#46	188890 (85.8)	31145 (14.2)	
**Urban/rural**				
**Urban**	#16	505507 (90.0)	56143 (10.0)	<0.001
	#26	505197 (89.7)	57965 (10.3)	
	#36	467256 (82.7)	97985 (17.3)	
	#46	466947 (82.8)	97320 (17.2)	
**Rural**	#16	592034 (89.5)	69158 (10.5)	<0.001
	#26	591845 (89.2)	71937 (10.8)	
	#36	543014 (80.7)	129936 (19.3)	
	#46	543236 (80.9)	128280 (19.1)	

*chi-squared test.

The prevalence of FPM caries in 2013 was 7.7% for tooth #16, 7.9% for #26, 14.1% for #36, and 13.8% for #46. In 2017, it increased to 12.4% for tooth #16, 12.8% for #26, 21.3% for #36 and 21.1% for #46. The differences in FPM caries rates from 2013 to 2017 were statistically significant (*p* < 0.001) (Fig 3).

Fig 1 shows that the prevalence of FPM caries in urban areas was 10.0% for tooth #16, with 10.3% for #26, 17.3% for #36, and 17.2% for #46. In rural areas, it increased to 10.5% for tooth #16, with 10.8% for #26, 19.3% for #36, and 19.1% for #46 (*p* < 0.001).

For teeth #16, #26, #36, and #46, caries prevalence rates were higher in the east (11.9% for tooth #16, 12.2% for #26, 21.4% for #36, and 21.3% for #46), the south (11.2% for tooth #16, 11.7% for #26, 20.2% for #36, and 20.1% for #46), the central region (9.5% for tooth #16, 9.8% for #26, 16.8% for #36, and 16.5 for #46) compared to the north (8.1% for tooth #16, 8.3% for #26, 14.4% for #36, and 14.2% for #46) and the west (7.4% for tooth #16, 7.5% for #26, 14.3% for #36 and 14.3% for #46) of Zhejiang Province. All the differences were statistically significant (p < 0.001). Fig 4 presents the percentages of caries in the four PFMs in terms of the geographic location of residence.

### The association between the relevant variables and FPM caries

The relationships between the relevant variables and caries risk (odds ratios, 95% confidence intervals) were calculated using multiple regression models (Table 4). It would be defined as prevalence of caries with at least one caries identified among the 4 studied molars. The female subjects exhibited a higher risk for FPM caries than the male subjects (OR = 1.38, 95% CI: 137–1.39). The OR values (95% CI) for the calendar year were 1.28 (1.26–1.31) in 2014, 1.21 (1.18–1.23) in 2015, 1.4 (1.37–1.43) in 2016, and 1.58 (1.55–1.62) in 2017 compared with those in 2013, indicating a general tendency of FPM caries to increase from 2013 to 2017. The central region of the province was taken as a reference. The eastern and southern regions were associated with an increased risk of caries; however, the western and northern regions exhibited a decreased risk of caries. Compared with children from urban areas, those from the rural areas had a higher risk of FPM caries (OR = 1.15, 95% CI: 1.14–1.16). Stratified analysis was performed in terms of gender. Irrespective of gender, the associations between calendar year, region, and rural/urban areas on the one hand and FPM caries, on the other hand, were very similar to those in the whole samples.

**Table 4:** The associations of relevant variables with caries risk.

Variable	Total (n = 1120569)		Boy (n = 586342)	Girl (n = 534227)
	OR (95% CI)	p	OR (95% CI)	OR (95% CI)
**Gender**				
Boy	Ref	--	--	--
Girl	1.38 (1.37~1.39)		--	--
**Calendar year**				
2013	Ref	--	--	--
2014	1.28 (1.26~1.31)	<0.0001	1.31 (1.27~1.35)	1.26 (1.23~1.3)
2015	1.21 (1.18~1.23)	<0.0001	1.22 (1.18~1.26)	1.19 (1.16~1.23)
2016	1.4 (1.37~1.43)	<0.0001	1.41 (1.37~1.45)	1.39 (1.35~1.43)
2017	1.58 (1.55~1.62)	<0.0001	1.58 (1.53~1.63)	1.59 (1.54~1.64)
**Region**				
Middle	Ref	--	--	--
East	1.35 (1.33~1.36)	<0.0001	1.36 (1.34~1.38)	1.34 (1.31~1.36)
South	1.3 (1.28~1.31)	<0.0001	1.31 (1.28~1.33)	1.29 (1.27~1.31)
West	0.81 (0.8~0.83)	<0.0001	0.82 (0.8~0.85)	0.8 (0.78~0.82)
North	0.82 (0.81~0.84)	<0.0001	0.83 (0.81~0.85)	0.82 (0.8~0.83)
**Urban/rural**				
Urban	Ref	--	--	--
Rural	1.15 (1.14~1.16)	<0.0001	1.15 (1.14~1.17)	1.15 (1.14~1.17)

*Multiple logistic regression model included all the variables above.

Additionally, the associations of gender, calendar year, region, and rural/urban areas with FPM caries in the whole samples and two genders were re-estimated. Table 5 presents the results in detail.

**Table 5:** The associations of relevant variables with caries risk in different tooth location.

Teeth	Variable	Total (n = 1222842)		Boy (n = 644248)	Girl (n = 578494)
		OR (95% CI)	p	OR (95% CI)	OR (95% CI)
**#16**	**Gender**				
	Boy	Ref	--	--	--
	Girl	1.35 (1.34~1.37)	<0.0001	--	--
	**Calendar year**				
	2013	Ref	--	--	--
	2014	1.29 (1.26~1.33)	<0.0001	1.31 (1.25~1.36)	1.28 (1.23~1.33)
	2015	1.2 (1.17~1.24)	<0.0001	1.21 (1.16~1.27)	1.2 (1.15~1.25)
	2016	1.41 (1.37~1.45)	<0.0001	1.42 (1.36~1.48)	1.4 (1.35~1.46)
	2017	1.66 (1.61~1.71)	<0.0001	1.64 (1.56~1.72)	1.68 (1.61~1.75)
	**Region**				
	Middle	Ref	--	--	--
	East	1.7 (1.67~1.74)	<0.0001	1.74 (1.69~1.79)	1.67 (1.63~1.72)
	South	1.53 (1.5~1.56)	<0.0001	1.55 (1.51~1.6)	1.51 (1.47~1.56)
	West	0.74 (0.72~0.76)	<0.0001	0.76 (0.73~0.8)	0.72 (0.69~0.75)
	North	0.84 (0.82~0.86)	<0.0001	0.86 (0.83~0.89)	0.83 (0.8~0.86)
	**Urban/rural**				
	Urban	Ref	--	--	--
	Rural	1.11 (1.1~1.13)	<0.0001	1.12 (1.1~1.15)	1.11 (1.09~1.13)
**#26**	**Gender**				
	Boy	Ref	--	--	--
	Girl	1.35 (1.34~1.37)	<0.0001	--	--
	**Calendar year**				
	2013	Ref	--	--	--
	2014	1.3 (1.26~1.33)	<0.0001	1.29 (1.24~1.35)	1.3 (1.25~1.35)
	2015	1.22 (1.18~1.26)	<0.0001	1.21 (1.16~1.26)	1.23 (1.18~1.28)
	2016	1.41 (1.37~1.45)	<0.0001	1.4 (1.34~1.46)	1.41 (1.36~1.47)
	2017	1.66 (1.61~1.72)	<0.0001	1.64 (1.57~1.72)	1.69 (1.62~1.76)
	**Region**				
	Middle	Ref	--	--	--
	East	1.71 (1.68~1.74)	<0.0001	1.74 (1.7~1.79)	1.68 (1.63~1.72)
	South	1.56 (1.53~1.59)	<0.0001	1.57 (1.53~1.62)	1.55 (1.51~1.59)
	West	0.73 (0.7~0.75)	<0.0001	0.75 (0.72~0.79)	0.7 (0.68~0.73)
	North	0.86 (0.84~0.88)	<0.0001	0.86 (0.83~0.89)	0.85 (0.82~0.88)
	**Urban/rural**				
	Urban	Ref	--	--	--
	Rural	1.11 (1.09~1.12)	<0.0001	1.11 (1.09~1.14)	1.1 (1.08~1.12)
**#36**	**Gender**				
	Boy	Ref	--	--	--
	Girl	1.42 (1.41~1.43)	<0.0001	--	--
	**Calendar year**				
	2013	Ref	--	--	--
	2014	1.29 (1.26~1.31)	<0.0001	1.3 (1.26~1.34)	1.27 (1.23~1.31)
	2015	1.22 (1.19~1.24)	<0.0001	1.22 (1.18~1.26)	1.22 (1.18~1.25)
	2016	1.43 (1.4~1.46)	<0.0001	1.44 (1.39~1.49)	1.42 (1.38~1.47)
	2017	1.61 (1.57~1.65)	<0.0001	1.61 (1.55~1.67)	1.61 (1.56~1.66)
	**Region**				
	Middle	Ref	--	--	--
	East	1.79 (1.76~1.82)	<0.0001	1.81 (1.77~1.85)	1.77 (1.73~1.81)
	South	1.55 (1.52~1.57)	<0.0001	1.56 (1.53~1.6)	1.53 (1.5~1.57)
	West	0.75 (0.74~0.77)	<0.0001	0.76 (0.73~0.79)	0.75 (0.73~0.77)
	North	0.88 (0.86~0.9)	<0.0001	0.88 (0.85~0.9)	0.88 (0.86~0.91)
	**Urban/rural**				
	Urban	Ref	--	--	--
	Rural	1.13 (1.12~1.14)	<0.0001	1.14 (1.12~1.16)	1.12 (1.1~1.14)
**#46**	**Gender**				
	Boy	Ref	--	--	--
	Girl	1.41 (1.4~1.42)	<0.0001	--	--
	**Calendar year**				
	2013	Ref	--	--	--
	2014	1.29 (1.26~1.32)	<0.0001	1.33 (1.28~1.37)	1.27 (1.23~1.31)
	2015	1.23 (1.2~1.26)	<0.0001	1.25 (1.21~1.29)	1.21 (1.17~1.25)
	2016	1.45 (1.42~1.49)	<0.0001	1.48 (1.43~1.53)	1.43 (1.39~1.48)
	2017	1.62 (1.58~1.66)	<0.0001	1.64 (1.59~1.71)	1.6 (1.55~1.66)
	**Region**				
	Middle	Ref	--	--	--
	East	1.81 (1.78~1.83)	<0.0001	1.82 (1.78~1.86)	1.79 (1.75~1.83)
	South	1.57 (1.54~1.59)	<0.0001	1.58 (1.55~1.62)	1.55 (1.52~1.58)
	West	0.77 (0.75~0.79)	<0.0001	0.77 (0.75~0.8)	0.76 (0.74~0.79)
	North	0.88 (0.87~0.9)	<0.0001	0.88 (0.86~0.9)	0.88 (0.86~0.91)
	**Urban/rural**				
	Urban	Ref	--	--	--
	Rural	1.12 (1.11~1.13)	<0.0001	1.14 (1.12~1.16)	1.1 (1.08~1.12)

*Multiple logistic regression model included all the variables above.

## Discussion

This study examined the prevalence of caries in first permanent molars (FPMs) in 6–8-year-old children in Zhejiang Province and their distribution in terms of period, gender, urban and rural areas, and geographic location of residence, i.e., south, north, east, west, and the central region. Almost all data on FPMs were included in 2013–2017, and the results are quite stable. There were no change in the inclusion process of children during this period or in the collection of the data.

It has been confirmed that the caries process in the FPM starts as soon as they erupt, and caries can be clinically observed within 1–2 years after eruption [13]. In this research, the data showed that the prevalence of FPM caries in Zhejiang Province followed a generally increasing trend in 6–8-year-old schoolchildren, with the incidence rising from 20.4% in 2013 to 29.0% in 2017, consistent with the results of the 4th CNOHS conducted in 2015–2016 [3]. With pit and fissure sealing conducted, it seemed that the caried prevalence rate would decrease. But in fact, efforts made to those children last year would not make sense to the children next year. The increasing trend just reflected the situation of the second grade children of different each year. The potential reasons for the increasing rate of FPM caries over the past five years in Zhejiang Province are complex since many factors might contribute to this trend.

The first important plausible explanation for this increase is the persistent lack of public awareness about oral health [14]. According to the results, 78.7% of the students had fully erupted FPMs at 6–8 years of age. However, some children and their parents were unaware of the existence of FPMs, which is consistent with the results of other studies [13]. The second explanation for this finding is the dietary pattern [15]. Over the past 20 years, dietary patterns in China have changed from a traditional diet to a modern diet with a high intake of meat, refined grains, and sugar-sweetened beverages among Chinese children aged 6–14 years [16]. The last but not the least plausible explanation is limited access to dental health services. A dental visit might ensure that the dentist would take preventive measures, such as the application of fluoride and fissure sealants, provision of oral hygiene instructions, and emphasis on the importance of regular visits to a dentist.

This investigation showed that the female schoolchildren had much higher caries prevalence compared to the male subjects (OR = 1.38). Evidence from ethnographic and epidemiological studies of dental caries in living human populations, as well as documentation from prehistoric human skeletal populations, shows that females usually, but not always, have a higher prevalence of dental caries [17]. Higher caries prevalence among females is often explained by both biological (genetics, hormones, and reproductive history) and anthropological (behavioral) factors, such as culture-based division of labor and gender-based dietary preferences [18].

According to the results, the caries prevalence of FPM in 6–8-year-old students in Zhejiang Province was 24.3% and 26.9% in urban and rural areas, respectively. Second-graders in rural areas were 1.15 times more likely to have PFM caries compared to those in urban areas (*p* < 0.0001), consistent with studies by Matilla et al. (2000) [19], Du Minquan et al. (2007) [20], and Jain et al. (2015) [21]. According to the results of China Statistical Yearbook (http://data.stats.gov.cn/publish.htm?sort=1, the per capita sugar consumption of rural households in Zhejiang Province increased from 1.23 kg/person in 2013 to 1.5 kg/person in 2015 and 1.8 kg/person in 2017, while the per capita sugar consumption of urban households has remained at 1.4 kg/person since 2015, which may due to the poor knowledge, the negative attitude and the improper behavior of oral health in rural families. More sugar has been added to drinks, cakes, snacks, and other favorite foods of children, further increasing children’s implicit sugar intake.

In addition, the lower use of toothpaste or toothbrushes [21, 22], as well as the lack of conventional dental care such as brushing teeth twice a day, using dental floss etc [23], might give rise to a higher FPM caries rate in children in rural areas, suggesting that these children will be the main population in need of oral disease preventive programs in China in the future.

As for the geographic region, the eastern and southern regions are at a higher risk, while the western and northern regions are at a lower risk for FPM caries in children compared to the central region of Zhejiang Province. As we all know, dietary fibers are significant nutritional factors affecting total caries experience and occlusal caries experience [24]. Generally speaking, the dietary pattern of residents in coastal areas is mostly similar to the Western dietary pattern (i.e., high in red meat, with poultry, organs, eggs, and seafood). The dietary pattern of those living in the open plains is similar to the traditional southern Chinese dietary pattern (i.e., high in refined grains, with vegetables and fruits); and the dietary pattern of those living in the hills consists of grains and vegetables (i.e., high in whole grains, with tubers and vegetables) [11]. The dietary pattern in the mountainous areas (west and part of the north and south) of Zhejiang Province is rich in high-fiber foods, such as bamboo shoots, vegetables, and nuts, which require a lot of chewing and are beneficial for caries prevention. At the same time, these findings are similar to those of the epidemiological survey of FPM caries in Guangdong Province of China [25]. Therefore, Zhejiang residents in the coastal areas and open plains and those accustomed to the Western dietary pattern should not only control the intake of sugar but also increase the intake of high-fiber foods in order to reduce the caries rate in children.

Based on the results of the present research, caries prevention strategies should focus on three aspects, which are also available for consultation beyond Zhejiang province in China. First, oral health education should raise awareness about the importance of oral hygiene and proper nutrition, particularly to limit sugar intake and soft non-abrasive foods. Second, the preventive programs should expand their target groups to cover a wider age range to include younger children, potentially beginning with prenatal education of mothers and adoption of proper measures. This investigation showed that in 2013–2017 over 20% of 6–8-year-old students had already developed FPM caries. The results suggested that the timing of the current OHPP misses the crucial developmental period before the initiation of FPM caries. The last but not the least is the emphasis on the leading role of the government in the prevention and management of oral diseases. In rural areas, most children’s dental problems cannot be managed, resulting in the loss of teeth at a very young age. They will not visit a clinic for restorative treatments due to defects in their dentition and will not adopt preventive measures in advance. Improvements in the economic status will give rise to more attention to oral health status. Unfortunately, oral health education has not been rendered in most rural areas. In such a case, the government-guided oral health education (OHE) can effectively control and prevent oral diseases. It includes the construction of a network for prevention, the management of oral diseases at all levels, and the establishment of national and regional stomatological/dental health education centers, effectively promoting the oral health during the whole life span.

### Limitations

The main limitations of the present study are: 1) the lack of a survey for oral health habits, diet, and family socioeconomic status; 2) the unavoidable shortcomings of the available data and their incompleteness; 3) the variability in diagnosis even with calibration, indicating that any conclusions that could be drawn might be speculative.

Further investigation should avoid these shortcomings in order to determine the factors contributing to the higher rate of FPM caries in students in Zhejiang Province.

## Conclusions

The trend towards a higher prevalence rate of FPM caries continues in Zhejiang Province. Female second-graders exhibited significantly more FPM caries experience than their male counterparts. Children in rural areas and the eastern and northern regions of Zhejiang Province had a higher FPM caries rate compared to those in the urban areas and the western and southern regions. The government should allocate a more generous budget to oral health education for children in rural areas. The prevalence of dental caries and its distribution in the population should be among the primary considerations in future planning for dental health prevention programs.
